# Social, Economic and Overall Health Impacts of COVID-19 on People Living with Disabilities in King County, WA

**DOI:** 10.3390/ijerph191710520

**Published:** 2022-08-24

**Authors:** Nicole Turcheti, Amy A. Laurent, Christina Delgado, Kayla Sainati, Kris Johnson, Eva Y. Wong

**Affiliations:** 1 Public Health—Seattle & King County, 401 5th Ave, Seattle, WA 98104, USA; 2 Disability Empowerment Center, 1401 E Jefferson St, Seattle, WA 98122, USA

**Keywords:** COVID-19, health disparities, people living with disabilities, health equity, social, economic, qualitative study, community engagement

## Abstract

The COVID-19 pandemic and the associated mitigation measures to reduce the spread of disease affected the social, economic, and overall health of individuals. Quantitative administrative datasets typically did not contain demographic information that allowed for reporting or analysis of the impacts of COVID-19 on people living with disabilities. Understanding the experiences of this population during the pandemic can inform the design of public health responses that are more robust and better connected to community. This paper describes a qualitative participatory study with a diverse sample of people living with disabilities in King County, WA. Through 2 listening sessions and 35 semi-structured interviews, it examines what impacts COVID-19 brought for people living with disabilities; elucidates the supports that were helpful in addressing COVID-19 impacts; examines inequities faced by the disability community; and sheds light on how to engage with this community to inform the public health emergency response. The process, protocols, findings, and lessons learned are replicable by other local health departments and could be incorporated as part of routine data collection and considered for future public health emergencies.

## 1. Introduction

The ongoing COVID-19 pandemic, as well as individual and community level mitigation strategies to reduce disease, affected the social, behavioral, economic, and non-COVID-19-related health outcomes. These impacts extended beyond the period of the mitigation policy and the effects on different populations were experienced in disparate ways [1]. Starting in May 2020, Public Health–Seattle and King County (PHSKC), the local public health agency for King County, Washington, leveraged the Centers for Disease Control and Prevention (CDC) Community Mitigation Strategy framework [2] to create a local project monitoring timely data to: (i) understand the impacts of non-pharmaceutical interventions (NPIs) mitigation approaches; (ii) inform public health policy and community decision making; and (iii) monitor community-level disparities that may occur during the pandemic [1]. Monitoring and addressing health disparities—i.e., differences in risk factors, health outcomes, and access to services—is a fundamental principle of PHSKC [3]. 

PHSKC’s COVID-19 impact monitoring project primarily used administrative datasets compiled from traditional and non-traditional public health sources, many of which cover the 2.3 million people who reside in the county. Most of these datasets have either zero or limited information about some of the populations who were particularly vulnerable to adverse COVID-19 outcomes, such as the population of people living with disabilities. To address this lack of data, PHSKC partnered with two local community-based organizations (CBOs) that serve people with all disability types, the Disability Empowerment Center [4] and the Lifelong Aging and Disabilities Services [5], to conduct a qualitative study to bring attention to these individuals’ lived experience.

During the collaboration to co-design a process to answer the question of what were the impacts of COVID-19 for people with disabilities, the community-based organizations raised the need to disseminate information about the importance and the process of how to engage with the disability community to inform policy. About one in four adults nationally [6] and 18% of the adults in King County [7] report some type of disability, creating a large constituency in need of representation in data reporting. The terminology “people with disabilities” covers a diverse group of people with a wide range of needs and abilities. Disability “types”—often classified into broad categories such as cognitive, hearing, mobility, vision, self-care, and independent living [8]—are not mutually exclusive. The individuals with the same type of disability can be affected in very different ways. Some of the disabilities may not be visible.

The people living with disabilities are more likely to have underlying health problems [9,10], which have been shown to increase the risk of COVID-19 infections [11,12]. In particular, individuals with intellectual disabilities have a higher risk of mortality from COVID-19 than the general population, even after controlling for comorbidities [13]. Having an intellectual disability is the strongest independent risk factor other than age for COVID-19 mortality [14]. The individuals who are immunocompromised are at a high risk of negative COVID-19 outcomes, even subsequent to vaccination [15,16,17]. The individuals with disabilities were also more likely to have their employment impacted by the COVID-19 pandemic and were more frequently laid off or dismissed from their job compared to the individuals without disability [18].

In addition to demonstrating how the people with disabilities are at a higher risk of adverse outcomes from COVID-19 and discussing the impacts on their employment, the recent literature also explores the impacts of the pandemic regarding issues such as transportation, mental health, and community participation for certain types of disability [19,20,21]. While the existing studies concentrate on COVID-19 impacts for a particular type of disability, or look at a single or a small set of types of such outcomes (e.g., employment, mental health), this study provides a broad overview of the different types of social, economic, and health consequences of COVID-19, contributing to understanding the intersections amongst them for the people living with disabilities. This study also examines what was helpful to support the disability community to deal with the negative impacts of the pandemic and the mitigation measures. Moreover, although the data about the impacts of COVID-19 for people with disabilities on the national level can provide insights, it is not representative of the experience of individuals with disabilities at the local level. The COVID-19 response and the resources made available to communities varied across states and localities [22,23], and therefore so did the impacts of these policies, and the pandemic in general. This study contributes to understanding these unique impacts for people with disabilities in King County, WA.

In short, this article: (i) answers the question of what impacts COVID-19 brought for people living with disabilities in King County; (ii) highlights how these impacts differed for this population; (iii) discusses what supports can be helpful to address the COVID-19 impacts and the barriers faced to access them; (iv) discloses other important issues raised by community members, such as inequities faced by the disability community and the need to systematically collect data about this group; and (v) sheds light on how to engage with this community to inform the public health emergency response by describing the study’s methods and lessons learned.

## 2. Materials and Methods

The qualitative data collection for this study was designed to align with the social, economic, and overall health categories established in the PHSKC framework (Table 1). Most of the available quantitative data regarding impacts of COVID-19 did not include information on ability status [24]. 

### 2.1. Study Design

**Figure 1:**
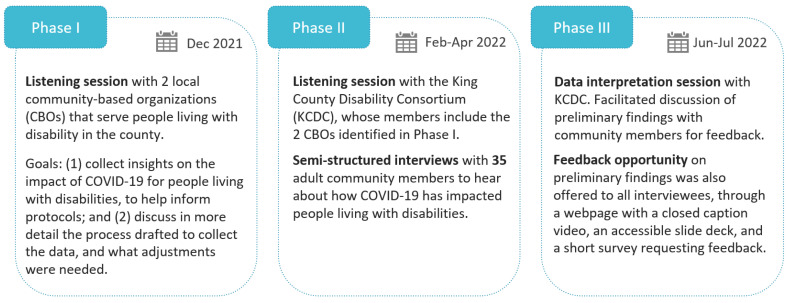
Data collection phases.

The data collection occurred in three primary phases, starting in December 2021 (Figure 1). The listening session in Phase I developed a new relationship with two CBOs providing services to people with disabilities to: (i) collect data on the impacts of COVID-19 for people with disabilities, which would help inform interview question design; and (ii) to discuss the process of data collection and address context and cultural appropriateness. Based on the CBOs’ input, PHSKC developed a data collection tool that included open-ended questions and quantitative questions adapted from other COVID-19 surveys. This tool (see Supplementary Materials, File S4) guided the semi-structured interviews with 35 community members recruited in Phase II. Phase II also included a listening session with the King County Disability Consortium (KCDC), a group composed of community members and representatives of different organizations that serve people with disabilities. In Phase III, PHSKC brought the analyses and findings back to the community members to review, validate themes and findings, and to provide another opportunity for data gathering for the topics that may have been missed in Phase II.

### 2.2. Qualitative Interviewer Training

The social research scientists from PHSKC held training sessions for the eight interviewers selected by CBOs. The sessions focused on informed consent, interview techniques, social determinants of health, question prompts, ensuring confidentiality, and how to navigate and use the data collection tool—which was programmed in Phonic [25], a web-based interview tool that records the quantitative and qualitative questions together. The interviewers practiced conducting the semi-structured interviews with each other, asked clarifying questions, and provided feedback on the questions and on implementation. Based on the interviewers’ feedback, PHSKC adjusted some of the interview questions. The interviewers used recorders for the in-person sessions and used webinar software to conduct and record remote interviews. PHSKC performed quality control during the first week of interviews by reviewing the transcripts and offering another interviewer training session that provided opportunities for additional learning and prompts. The interviewers had a range of experience with previous interviews, and some spoke other languages. All of the interviewers signed an interviewer confidentiality agreement, shown in Supplementary Materials, File S1.

### 2.3. Recruitment and Data Collection

#### 2.3.1. Interviews

While the goal of this study was not to be able to generalize findings to the larger community of people living with disabilities, PHSKC and partner CBOs sought to ensure that the sample of interviewees had some representation from each of the five major disability categories that were suggested by the CBOs: (i) mental health condition; (ii) developmental or intellectual disability; (iii) mobility disability; (iv) sensory disability; and (v) other disability or chronic condition. The sampling frame consisted of people receiving services through the partner CBOs and the University of Washington School of Social Work students identifying as living with disabilities. The sampled participants were those available during the two-week data collection period. Of the 35 interviews conducted, 26 were with clients and 9 with students. Eight of the CBO staff conducted the interviews. After the first week of interviews, PHSKC reviewed the types of disabilities self-identified by the participants, and the CBO leads included additional people in the sample to ensure representativeness. The participants received USD50 for their time, presented as cash or gift card options. The interviews occurred either online or in person with appropriate infection controls for the in-person interactions during April 2022.

During the pandemic, many of the previous in-person interactions transferred to a digital or online experience. However, for some of the individuals living with disability, technology could have been challenging to use because of their type of disability or medical condition (e.g., brain injury), lack of appropriate devices, low digital literacy, or lack of high-speed internet. Given the potential for COVID-19 infection concerns, PHSKC and the partner CBOs worked to strike a balance between the risk of in-person interviews versus missing data collection opportunities for individuals who were not able to use the technology solutions. Some of the interviewers were already seeing clients in their home or in a clinic setting and so were using appropriate infection controls for in-person interactions. PHSKC also worked with the CBOs to develop a process by which an interviewee could call into a webinar line, and answer questions to address issues with the people who were uncomfortable or unable to be in-person, but who had technology limitations. The CBOs had sign language interpreters who were available for interviews. The interviewees orally shared their consent at the beginning of the data collection session.

#### 2.3.2. Listening Sessions

The Phase I and Phase II listening sessions (as well as Phase III data interpretation session) occurred during regularly scheduled online KCDC meetings, with live transcription services, a sign language interpreter, and a PHSKC note-taker. The participants orally shared their consent prior to listening sessions. The topics discussed in the Phase I and Phase II listening sessions are in Supplementary Materials, File S2 and File S3, respectively.

### 2.4. Analysis 

The quantitative and qualitative data collected through Phonic were downloaded. The qualitative responses were automatically transcribed by the software and checked for accuracy, using the audio files from the backup recordings completed by the interviewers. The audio files from the in-person interviews and the recordings from webinar software were saved, checked for accuracy, and transcribed by PHSKC. For the listening sessions, the data were captured by the PHSKC note-takers and recorded through the webinar software used by KCDC to host their regular meetings. The qualitative findings were analyzed using an inductive thematic approach [26]. The PHSKC team began developing a codebook from the ideas discussed with CBOs in Phase I. Three experienced coders reviewed the listening sessions notes and interview transcripts to refine the codebook, using the notes to document thematic evolution. The themes were established and coded using Dedoose [27]. The coders participated in inter-rater reliability testing after a group review of the themes and codes for consistency in coding. The initial results indicated moderate agreement among the coders (average pooled Cohen’s Kappa = 0.55) [28,29]. A second group training session was set up to increase the reliability amongst the coders, and included a review of the coding differences and refinement of the analysis codebook. The coders checked with each other frequently during the coding process to support consistent coding.

## 3. Results

### 3.1. Demographics of Interviewees

Most of the individuals were between 25–34 years of age, female, white, and not previously infected with COVID-19 (Table 2). The different types of disability groups were well represented, as many of the individuals self-selected more than one disability type. The sample had a larger percent of individuals identifying as non-binary/genderqueer and transgender as compared to the overall population. Most of the people in the sample (91%) self-identified as having a mental health disability, which is higher than the percentage of the Disability Empowerment Center’s clients who identify as having a mental health disability (37%) [30]. The participants also had slightly higher rates of vaccination followed by booster (52% for the overall King County population) [31].

### 3.2. Findings

The findings from the listening sessions and semi-structured interviews were organized in the following groups: (i) impacts of COVID-19 for people living with disabilities; (ii) supports to deal with such impacts; and (iii) other themes that relate to inequities experienced by this community and highlighted by the COVID-19 pandemic. The results show that the vast majority of interviewees experienced adverse impacts from COVID-19.

#### 3.2.1. Impacts of COVID-19 for People Living with Disabilities

##### Work/Employment

COVID-19 impacted work or employment situations for 21 out of the 35 interviewees. The individuals reported having to quit work, change jobs, or shift to working from home due to the need to protect against COVID-19. One interviewee shared:

“*Because I’m immunosuppressed, at high risk for COVID, I decided to just work online. And that really impacted the hours that I’m working. I’m working less because of that. They don’t have a lot of the online work for me to do. Not as much as when I was working in-person*”.

*(Interviewee)*

Others noted that they had work hours cut, were furloughed, or had their workplace close because of COVID-19. Some of the participants talked about their own experience or that of other people with disabilities in their community not being able to find work for months after losing their job. One participant noted: “*when the pandemic first started, I was working retail and so we shut down entirely; and so, I was unemployed for a good six months at [the] start of pandemic*” *(Interviewee)*.

The COVID-19 pandemic exacerbated the pre-existing challenges that the people living with disabilities face in obtaining a job [17]. The participants talked about the increased mental health issues related to COVID-19 leading to the loss of their job:

“*… it’s been pretty hard to maintain employment because of what’s going on with my mental health. At one point during the pandemic, I was working [an] on call job and it was really bad for my mental health, so I actually had to leave the job. So, [for] most of the pandemic, I have not been working*”.

*(Interviewee)*

The participants also identified the positive impacts of the pandemic on their work situation, such as receiving more flexibility from employers (e.g., being allowed to work from home), or having more work opportunities available with the increase in telework job opportunities:

“*I will add that some positives that came out of it—that people who have disabilities … would have liked to have seen adapted a lot faster that [the] pandemic made possible—was the push towards remote work. I don’t drive or anything like that. So, going into work I was always very limited … Now that there are so many options out there that are purely remote that gives me a lot more opportunity to be able to find employment*”.

*(KCDC member)*

##### Finances

The struggle to pay rent and bills, as well as accumulating debt, were experiences shared by many of the participants. Overall, 28 of the 35 interviewees reported financial impacts of the COVID-19 pandemic. Having to rely on friends, family and/or financial aid to manage during the pandemic was commonly reported. The negative impacts on work/employment worsened the financial situation of some participants:

“*… with my lack of job stability because of the pandemic and not having employers that are aligned with my safety standards … I’ve racked up I think almost $10,000 in debt and credit card debt being in between jobs and needing to support my family and pay rent*”.

*(Interviewee)*

Medical expenses often reinforced the reported financial strain, as exemplified by a participant who shared: “*I’ve spent significantly more money in the pandemic on medical expenses and living than I have any other time …*”* (Interviewee)*. Others also faced increased expenses from having food delivered at home since they were homebound for safety. Some shared that they were not able to afford medical supplies or needed mental health services:

“*I also had like a tragedy, a personal tragedy happened during the pandemic … a suicide in the family and that really deeply impacted me, and I was not able to get treatment for that … To be able to get treatment for that would have also been paying out of pocket … I wasn’t able to get … grief counseling or anything really that I needed for that*”.

*(Interviewee)*

The improved financial support programs were a type of NPI that had positive impacts, according to the participants:

“*I was already not receiving, not getting any money and it [COVID-19] made it easier for me to get ADB [Aged, Blind or Disabled Cash Assistance]. That was something that was a relief. People were more giving of money than they had been in the past because they knew that everyone was going through something*”.

*(Interviewee)*

##### Food Security

Of the 35 people interviewed, 21 reported having a time when their food did not last and they did not have any money to buy more. Ten of these 21 people had not had a similar situation before the pandemic. In addition, amongst the 35 interviewees, 14 had someone in their household eat less than they felt they should because there was not enough money to buy food; and of the 14 people, 9 had not had a similar situation before the pandemic.

One interviewee shared:

“*I’m starving like about a week and a half, two weeks [per month]. …[on] the last week of the month I’m totally broke. I don’t have no food. You know, it’s just crazy … It’s just hard to survive off of what I received from SSI [Supplemental Security Income]*”.

*(Interviewee)*

Another participant also shared that the benefits were insufficient to ensure food security:

“*Food has been so expensive. They increased the food stamp amount to the maximum but that didn’t even last a month. Then the rest of the month, I had to rely on food banks, but food banks aren’t getting the donations that they used to, so they’re short on food and yeah, it’s been a real struggle. There have been times when I lose weight, because I’m forced to eat only one meal a day because I can’t afford to eat more than that*”.

*(Interviewee)*

##### Housing

Almost half of the interviewees (15) reported having their housing situation impacted by COVID-19. The increased rent prices was a major theme, sometimes meaning that people living with disabilities had to move to a different home:

“*Another impact [of COVID-19] … is that I [had] to move. I’m not really going to be living in Seattle that much longer. Just because the cost of living has … They’re raising my rent … And so it’s just not really working for me living here. So, I’m having to uproot my life now, which is really stressful*”.

*(Interviewee)*

In addition to having to move or choosing to move to a different home, participants mentioned applying for rent assistance programs, as well as delaying rent or mortgage payments. Some of the participants also described feeling concerned about being evicted from their homes, and others shared that they ended up experiencing homelessness: “*I was homeless for much of the pandemic*” *(Interviewee)*. On the other hand, another participant said that, thanks to the increase in available supports during the pandemic, they were able to leave their condition of homelessness:

“*It’s made me have a house. I think that one of the things that was sad for so many people. So many people lost a lot. But for the homeless, I think it helped a lot of people get housed*”.

(*(Interviewee)*

##### Mental Health

The mental health impacts were the most commonly reported outcome in this study. Amongst the 35 interviewees, 34 indicated their mental health had been impacted by the pandemic. Many of the participants shared accounts of worsening anxiety and depression. Two interviewees shared:

“*I think the main thing I’ve noticed in the pandemic is particularly around mental health, that’s where I feel it the most. The longer I’ve been in the pandemic I just feel like it really adds to burnout and everything else going on and so they’re just really—there are days where it’s just really hard to get up in the morning …*”

*(Interviewee)*

“*COVID … has impacted my lifestyle pretty severely. I have virtually no social life … I do deal with anxiety, and it has been getting worse and worse, and I’m trying to manage it. But the restrictions because of COVID has [sic] made my anxiety quite a bit worse and I am sensitive to the anxiety around me, which is everywhere I go. And it’s, you know, so yeah, the atmosphere of mistrust, the atmosphere of anxiety with a lot of people. I think the anxiety over … I mean, a lot of people are very anxious about the economy, and you know, there’s just a lot of that so that impacts me pretty strong*”.

*(Interviewee)*

The mental health impacts reported by interviewees included feeling anxious or depressed more often (*n* = 33); having less healthy habits, e.g., eating too much, smoking, drinking, using drugs or other substances (*n =* 27); losing access to counselor or support system (*n =* 17); and losing continued access to necessary medication for mental health (*n =* 9). A participant shared: “*I had anxiety and worry too much. I could not sleep. I started eating continuously*” *(Interviewee)*.

Others expressed feelings of social isolation, concern about contracting COVID-19, and accounts of the pandemic worsening the existing mental health conditions. Additional themes included being affected by their child’s worsening mental health state, being in a toxic relationship and unable to move out because of the pandemic, feeling overwhelmed by all of the changes in their life, and not being able to engage in activities that used to bring joy (e.g., meeting friends, taking fitness classes, or going to church).

An Interviewee explained:

“*it’s definitely affected my mental health. … my PTSD has been skyrocketing, my anxiety levels have been skyrocketing. I feel like kind of paralyzed a lot of the time now, like, just in my day-to-day life, I just feel very like paralyzed with anxiety. … because I don’t really have much stability in my life right now. … I don’t really feel like I have a sense of security anymore, which is a pretty bad feeling*”.

*(Interviewee)*

The feelings about mental health services shifting to virtual were split: some considered it a negative impact, as it created a barrier for them to access services; others considered it a positive change, as it made it easier for them to obtain mental health care. A total of 13 interviewees reported an increased access to a counselor or support system. An interviewee shared that: “*In some ways that’s helped me lean forward into getting more mental health help so I’m more resilient and capable to handle things on my own*” *(Interviewee)*. Another positive impact, reported by 11 interviewees, was starting to have healthier habits, such as eating better, exercising, and/or making more time for hobbies.

Finally, some of the participants appreciated that the pandemic brought more awareness to the importance of mental health. A member of KCDC explained:

“*I think the pandemic has really shed a light on the importance of putting an emphasis on taking care of yourself and mental health and we still have a long way to go as a society. I think that companies are getting more to that point that yes you can take a mental health day*”.

*(KCDC member)*

##### Physical Health

Some people reported positive changes to their physical health, such as “*[I] quit smoking … [In] some ways it’s the only part of my life that I can have full control over to help me get through this, if that makes any sense*” (Interviewee). However, most of the reported impacts on participants’ physical health were negative. Half of the interviewees (17) avoided seeking health care because of concerns about becoming infected with COVID-19, and 20 had surgery or another medical procedure delayed because of the pandemic.

While the PHSKC framework was focused on non-COVID-19-related health outcomes, COVID-19 had other impacts on physical health. Some of the participants who were infected with COVID-19 had the disease worsen other health issues. For example, “*[Having COVID-19] aggravated the Crohn’s, which dovetailed into like blood clots in my lungs and it, like I say, it’s been an interesting almost 24 months for me, health-wise*” *(Interviewee)*. Finally, the participants shared how the measures to slow the spread of COVID-19 impacted their ability to keep healthy habits such as exercising—gym closures being a common example.

##### Physical and Emotional Safety

Physical and emotional safety emerged interwoven in five participants’ accounts of mental health, physical health and other impacts of COVID-19. One participant reported being sexually assaulted and pondering the increase of sexual violence during the pandemic:

“*I have felt anxious and depressed more often. I have had less healthy habits, eating less. Less exercise, less hobbies. I lost access to counselors and my support system. I lost access to medications for mental health as well as losing my therapist. I went through a sexual assault from someone that I had known for four years during the pandemic. I had that question from someone else a while ago and I found it interesting that sexual violence increased during the pandemic*”.

*(Interviewee)*

Other threats to physical and emotional safety in the data included experiences of home health caregivers “… *that have been bullies, dangerous, drugged me, done all kinds of stuff* …” (Interviewee), and an increased fear for physical safety while utilizing public transportation. Another participant spoke more broadly about the escalating domestic violence rates: “*I do want to say that for people who experience DV [domestic violence] the pandemic can be an especially hard time for them because there’s more people working from home and there’s this greater incidence of DV in general.*” *(Interviewee)*.

##### Access to Services

Accessing services to care for their health, including access to caregivers, medical providers, and medication, was common amongst the participants. One main sub-theme was feeling unsafe going to healthcare facilities because of the risk of exposure to COVID-19, which one participant argued was worsened by healthcare facility practices:

“*I have gone from going into the hospital at least once a week (often two times a week) to once every couple of months. Honestly, I’m terrified every time I go in. I have gone in and doctors were not wearing masks in the lobby … Waiting rooms are completely packed and no social distancing. I get terrified. Truly terrified*”.

*(KCDC member)*

The participants also discussed the overall decrease in availability of services, as healthcare systems were overwhelmed. They lost access to services or had health care procedures and/or services delayed. Some of the changes, such as telehealth, were reported as increasing access to services as a result of the pandemic.

##### Transportation

The participants reported feeling unsafe to take public transportation for fear of becoming infected with COVID-19. An interviewee shared: “*The bus. I hate it because I don’t know if I’ll catch COVID-19 or not*” *(Interviewee)*. Another participant echoed this sentiment, pointing out that not owning a car meant either increased risk of becoming infected or increased isolation:

“*Drive up and go shopping is great, if you have a car. If you don’t have a car, and you are at increased risk of contracting COVID, it may be too risky to take public transit, further increasing isolation*”.

*(KCDC member)*

This was exacerbated by a decrease in the available specialized transportation services for people living with disabilities, particularly in volunteer-based services. A KCDC member pointed out:

“*The domino effect in terms of stay-at-home orders and social distancing orders [is that] a lot of the clients we work directly with on my team are people with specialized transportation needs. People with disabilities, older adults, folks with limited mobility. What we saw and what we continue to see during the pandemic especially early on [is] a reduction in available services, which led to longer wait times, more uncertainty around a service being able to pick you up and drop you off where you needed, more anxiety and worry around safety while on those transportation whether it is a shuttle or individual’s car. Particularly volunteer-based services have been impacted and those are the services that in years previous have come highly recommended and were preferred because they offered a sense of community and connection to a lot of our clients … A lot of volunteers decided to stop driving for their own safety for their own health and wellness …*”

*(KCDC member)*

##### Education

According to the participants, it was challenging for children with disabilities—particularly those with an intellectual disability—to attend school virtually. It was stressful for caregivers to support children with disabilities in meeting their education goals during the pandemic. A member of the KCDC shared the opposing perspectives about the stay-at-home orders: on the one hand, they helped keep the community safe, but on the other hand, they brought hardship for families with children with disabilities. They explained:

“*I don’t think that the schools were equipped to help children with disabilities succeed; and with their IEP plans [Individualized Educational Plan] and putting that on the back burner and not giving kids their hours that are needed, and having that put on the parents even more to make sure their child is getting what they are owed while also even working or not having access to internet or a computer. So, I think there was a huge educational impact when it came to [mitigation stay-at-home orders]—and stay-at-home orders are great, and we should definitely stay home and be safe … I’m not sure if the schools could have done more to help families*”.

*(KCDC member)*

The participants also pointed out that the lack of support for primary caregivers increased their burden by placing more responsibility on them to meet their children’s education needs. Finally, some of the participants talked about delaying going back to school or fearing it, due to the risk of becoming infected with COVID-19.

##### How COVID-19 Impacts Differed for People Living with Disabilities

While the general public shared many of the impacts described above [1], some impacts were uniquely experienced by people living with disabilities—due not only to having a disability but also to the unaddressed inequities that existed before the pandemic, and to having insufficient support [11]:

“*COVID-19 impacts people with disabilities significantly differently because they are already at high risk for contracting, getting sick from, and dying from COVID-19. We all have different forms of care that we need and seek from the medical system, and when the system is overburdened and at capacity, people with disabilities lose their essential support and critical care that they need to survive, to live, to thrive*”.

*(Interviewee)*

Obtaining information about COVID-19 and the available resources was difficult for people with disabilities because communication pieces were not always produced in an accessible way. Accessing care was more challenging for this population because of the increased risk of exposure at healthcare facilities, accessibility issues at vaccination sites, caregiver scarcity, etc., as discussed above.

The increased isolation for those who live with a disability was also a common theme. For the individuals with certain types of disability (e.g., people with mobility restrictions or who are immunocompromised) it was harder or not advisable to go out. The other issues related to the stay-at-home orders augmented the isolation even further. A participant shared their experience of being unable to leave their home for two years because the replacement of their wheelchair battery was delayed due to COVID-19. Other participants talked about how using virtual platforms to deal with social isolation was not an option for people with certain types of disability. One participant shared: “*Many people have combated isolation by being on Zoom. As a person with a brain injury, screens are my enemy. Thus, resulting in more isolation and depression*” *(KCDC member).*

The participants pointed out that the routine activities, such as grocery shopping or using public transportation, brought a greater risk for those who are immunocompromised. Some shared they had no choice but to change or quit their jobs due to the higher risk of complications from COVID-19. For some, the financial burden that came from taking the necessary protective measures against COVID-19—e.g., wearing N95 masks and having groceries delivered—was a matter of survival and therefore was not optional, even if it meant accruing debt. Finally, the participants talked about masks making communication more challenging for those with a hard-of-hearing disability, and children with intellectual and developmental disabilities struggling to attend school virtually during the pandemic.

#### 3.2.2. Supports to Deal with Impacts of COVID-19

The participants shared their thoughts on the measures, resources and policies that supported, or could have supported, the disability community when confronting COVID-19-related issues. The supports include government-led initiatives, as well as initiatives led by community members.

##### Helpful Supports for COVID-19 Impacts

Receiving financial support, either from friends and family or from relief programs, was cited by many participants as providing meaningful assistance in weathering COVID-19. The stimulus checks were repeatedly upheld as a significant support “… *especially to those of us on SSI or SSDI [Social Security Disability Insurance]” (KCDC member).* Increases to other benefits brought “*… finally a livable amount of assistance*” *(KCDC member).* The participants illuminated the role that a robust social network of friends and family played in assuring a myriad of financial obligations—from rent, bills, internet service, to groceries—were covered.

For some of the recipients, receiving application assistance for the financial support programs was key. One interviewee credited their qualification for the rent relief program to the building manager, who applied for everybody in the building. Simplified processes, where applicants are not made to “*… jump through a million hoops …*” *(Interviewee)*, as one participant described, along with positive interactions with staff assisting in the program entry points contributed to successful enrollment.

The education sector was called out as a key connector to support. The school staff, such as a “success coach” or an “education advocate”, served as helpful guides in connecting potential recipients to programs and resources, in addition to helping students navigate COVID-19 changes. The academic institutions offered a range of direct support programs to their students, including monetary funds, internet support or hardware: “*I got a Macbook Pro through them*” *(Interviewee).* Another interviewee reported the college they attended “*… sent me two relief checks as part of my disbursement that was specifically for people being impacted by COVID-19*”* (Interviewee)*.

The participants shared that having continued access to a therapist during the pandemic served as a vital connection to the outside world. Some of the interviewees expressed feeling “*trapped*” and “*isolated*”, particularly due to a mobility disability, which presented additional challenges in engaging with the outside world. For some of the participants, the routine contact with a counselor met a need for social engagement. One participant shared: “*If I didn’t have caregivers and therapists my social life would’ve been pretty bleak*” *(KCDC member)*.

While the participants shared mixed experiences with health care overall, an attuned and responsive medical team was reported as having had a positive impact on a patient’s well-being. They were described as helpful, because they timely and proactively communicated COVID-related health information, such as vaccine availability. Finally, the participants relayed positive reactions regarding the ability of local organizations to adapt, altering service provision in creative ways, to effectively respond to community needs. The examples included volunteer organizations and specialized transportation services pivoting to food delivery, and grocery stores providing dedicated shopping hours.

##### Barriers to Accessing Resources

Some of the participants communicated a lack of awareness about the programs available to support them during the pandemic. Some knew about the programs but lacked clarity about how to connect to, or apply for, the services. Some expressed frustration with an overly cumbersome application process or failure to fully qualify for funding, due to certain exclusionary criteria. The exhaustion of current systems in place was captured by one participant, sharing their experience of seeking support while ill:

“*… fighting with insurance to get [medication]… yeah, that was not fun, and then fighting with the people for unemployment. And I’m sick during this period, I mean, it’s like insane, I should not have been dealing with all of that at once*”.

*(Interviewee)*

The interviewees also mentioned needing reduced barriers for accessing services, including information about COVID-19 and available resources (e.g., vaccination sites) produced accessibly. The examples included producing items accessible by print or mail for those who had a cognitive disability or lacked high speed internet.

##### Potential Improvements for COVID-19-Related Supports

The participants’ thoughts on how to improve the supports available during the pandemic included a more accelerated roll-out of programs coupled with a desire for greater frequency of dispersal. The participants appreciated the economic impact payments (or stimulus checks), but said they could have used additional support. A tight food budget led many participants to report that increasing the per household dollar amount for food stamps would be helpful in covering the actual expense of groceries, particularly given the influence of inflation on food costs. Recognizing the profound mental health toll of the pandemic, a desire for greater access to and visibility of free mental health services was a common sentiment expressed. More resources such as support programs or emergency shelter prioritization for people experiencing domestic violence were also common suggestions.

There was a call for government agencies to more effectively anticipate the unique needs of people living with disabilities and take them into account when designing supports. The examples included distributing free infection prevention supplies, recognizing the need for clear masks to aid in communication, developing accessible public health communication, and being cognizant of the benefit of affordable (or free) delivery of food and other essential products to populations experiencing greater barriers or risks to in-person transactions.

#### 3.2.3. Other Themes

##### Disenfranchisement

Disenfranchisement, i.e., the feelings of frustration with the inequities of not attending to the needs of the disability community to ensure folks are safe and healthy, was a common theme. The participants talked about people with disabilities not receiving “*reasonable accommodations at work, at school, at medical settings, and at other settings so that we can feel more safe, comfortable, and healthy*” *(KCDC member)*. Accessibility in vaccination sites was called out by participants as another example.

The participants expressed frustration and pointed out that insufficient attention to the needs of the disability community did not start with the pandemic, rather it was merely illuminated by it. They argued for the need to discuss “*What was broken before this [pandemic] in all of the delivery systems? In all of the services?*” *(KCDC member).*

Another reported reason for this feeling of frustration in the pandemic context was the lack of COVID-19-related data disaggregated by ability status. As described in a listening session:

“*… we are not collecting statistics on people with disabilities. When I got testing, never once did anyone ask if I had a disability. That is huge … You can’t recognize us as a community until you look at our data and collect data on us especially in this data-driven world. We are making funding decisions based on data. If we are not collecting information about us, then we are not included in those decisions or disproportionately unequally included*”.

*(KCDC member)*

Finally, the participants also talked about feeling “left behind” because of insufficient information about how COVID-19 impacted different types of disability. As exemplified by a participant: “*Folks that are immunocompromised are not the only people that is* [sic] *higher risk. People with intellectual disability are at higher risk for poorer outcomes. That information is not really out there*” *(KCDC member)*.

##### Reactions about Government Policies

Information was a theme also present in the context of government policies and actions regarding COVID-19. The participants argued that the government agencies failed to attend to the needs of the disability community, starting with not producing accessible information about COVID-19 and the resources available to deal with its impacts.

Some of the participants expressed discontent with lockdowns, as well as vaccination and the mask requirements in place. They pointed out that for the hard-of-hearing, for instance, wearing a mask can make communication more challenging, as it mutes sound and prevents lip reading, and that masks with clear mouths were hard to find. Other participants shared an appreciation of the policies and measures that helped protect the community against COVID-19, particularly the mask mandate and social distancing. According to them, it would be important to maintain these measures in order to help support people living with disabilities during the pandemic. One participant criticized the end of the mask mandate and messaging around it:

“*At the very least, please be transparent and actually tell the public the truth, that the mask mandate is not going away because it’s actually safer. … lots of people trust you [the WA state DOH] and follow the mask mandates, and they believe that the mask mandates will only be lifted when it’s safe, so therefore the mask mandates being lifted means it’s safe. People really believe that. But it’s not true. It’s not any safer. It’s just that the hospitals are less overwhelmed. Over and over again, cases start to go down, mask mandate gets lifted, then they climb back up, then the mask mandates go into effect again. People see it as inconsistency, and they lose belief and are less likely to follow mandates when they are in effect. At least tell people that they are not actually safer, it’s just that the healthcare system is ready/equipped for the next spike*”.

*(KCDC member)*

Other participants talked about their mistrust in government institutions, as exemplified by an interviewee who shared “*it [the response to COVID-19] made me very suspicious of world leaders and the government in general*” *(Interviewee)*.

Finally, some of the participants shared appreciation for the government responses during the pandemic, such as the eviction moratorium and the stimulus checks. There were also mentions about how the Washington state government did a better job than other parts of the country in supporting people during the pandemic.

##### Concerns about the near Future

Lastly, the participants shared concerns about what the future holds for people with disabilities. More specifically, they expressed worry about the end of the financial supports that were made available during the pandemic and how it will affect people living with disabilities, being that many were already struggling to live on the existing benefits prior to COVID-19.

An interviewee summarized the concerns about the future, contextualized by a broader sense of deprivation expressed by members of the disability community:

“*I’m very concerned about communities not bouncing back from this in the same way they were before. Like HIV/AIDS epidemic, when it first started, like where people were dying and the government wasn’t doing anything … they just didn’t care because they were considered like a disposable population*”.

*(Interviewee)*

## 4. Discussion and Lessons Learned

The findings from this study shed light on how the people living with disabilities in King County have experienced the impacts of the COVID-19 pandemic and the mitigation strategies taken to slow its spread, as well as the supports that were most helpful to address their negative effects. Notably, this study contributes to understanding the ways in which the COVID-19 impacts differed for people with disabilities, provides insights on engaging with the community to collect data and inform policy, and underscores the need for this critical population to be included in the demographics of routine data collection and dissemination. The recent literature also supports similar findings of more severe COVID-19 impacts on this population [32].

Although this study used the framework from PHSKC’s quantitative project to monitor the impacts of COVID-19, direct comparisons to such impacts for the general population cannot be made, due to the differences in data collection, time frame, and sampling. The results of this study suggest, however, that the impacts on the population of people living with disability were much more encompassing than in the overall King County population [24].

For example, 66% of those interviewed for this study reported that food did not last; a similar question in a convenience sample showed 15.3% of the overall population experienced this type of food insecurity [33]. Feeling anxiety or depression more than half the week peaked in the general population at 41% in January 2021. Amongst the 35 people with disabilities interviewed for this study, 94% reported feeling anxious or depressed more often during the pandemic [34]. Other national studies have found elevated anxiety and depression results in the community of people living with disability [35,36].

Collecting data disaggregated by disability status would have allowed for faster data processing, better comparison to understand differential impacts, and regular data updates, rather than a singular data collection and report—giving opportunities for public health, policy makers, and community leaders to leverage resources to reduce the negative impacts of the COVID-19 pandemic. In the absence of datasets that include information on the impacts of COVID-19 for people with disabilities, the study conducted by PHSKC in partnership with CBOs contributes to elevating the experience of this community. It also illuminates some of the implications these experiences have for the various system changes that need to occur to better serve people living with disabilities.

In particular, the study’s findings document the challenges in accessing information on COVID-19 or obtaining supports; thus, suggesting that attention should be given to the specific needs of people living with disabilities from the initial stages of a public health emergency. This may involve having preparedness plans with specific planning that make reaching this population a primary consideration. The participants pointed out the importance of having communication pieces (blogs, videos, social media live events) released in accessible formats (braille, with American Sign Language interpretation, live captioning, etc.) to inform the community about COVID-19, the measures to protect against it, and the economic and social supports that were made available to help mitigate the impacts. Similarly, ensuring accessibility to and within vaccination sites, and encouraging accommodations be made by businesses and organizations that provide essential services were specific call-outs from the participants as important supports for people with disabilities to better cope with the impacts of COVID-19. The examples of supportive accommodations include having dedicated shopping hours for people in higher risk groups, such as individuals whose disability is related to autoimmune conditions; seats for people who are waiting at vaccination sites; and large print information sheets. Accessibility that is lacking or inadequate can pose as a barrier to obtaining care and resources, as well as reinforcing existing inequities that people with disabilities currently face.

The participants talked about how one-off studies should not be the main or sole source of information about the impacts of COVID-19—or other health-related issues—for people living with disabilities. Ideally, this study would have been designed and implemented in the first year of the pandemic for an opportunity to adjust the processes more readily. The data about the impacts of COVID-19 for the general population were used in policy decisions and resource allocation for specific communities for which data were available [1]. In some cases, the study participants did not know about the programs and services that they would have been eligible to receive, so future outreach should involve improved information sharing. Earlier data collection could have been useful to address the missed opportunities for resources and connections, as well as to engage people with disabilities better in the pandemic response. Moreover, since this study asked the respondents to reflect back over the entire pandemic, nuance about the timing of policy interventions may have been blurred.

The limitations of this study also include the sample size, which was not large enough to generalize the results nor to fully explore intersectionality—i.e., the combined impact across social categories such as race, ethnicity, gender, sexual orientation, socioeconomic status, recognizing that the individual lived experience is multidimensional and the systems of privilege and oppression have influences across health outcomes [37]. Although the themes were becoming evident with the sample size of people interviewed, in the future, more interviews might be useful in understanding the nuances of intersectionality as they relate to public health emergencies and what the population is experiencing. The percentage of interviewees who reported a mental health condition was higher than reported by the clients from one of the partner CBOs. It is possible that the participants self-identified as having a mental health issue that impacted their daily lives without an official diagnosis or that some participants received a diagnosis during the pandemic. Finally, although this study highlights some of the aspects of the impacts of COVID-19 that are particular to certain types of disability, it falls short of being comprehensive of all the different ways that each type of disability influences how people experience such impacts.

There are some important insights that can be drawn from the participatory nature of this study regarding how to engage with the disability community to inform public health emergency responses. In order to do this work well, it is important to start collaborating with community in the early stages. Understanding the perspective of community partners, community members, subject matter experts, and groups providing services to individuals is valuable in developing the questions that address the issues that the community is experiencing. The relationship building with partners takes time but brings strengths and information not elsewhere available. The listening sessions during this study brought valuable feedback, built relationships, and worked to establish trust, along with providing validation of the results. In communities that have distrust of academic or governmental partners, building on existing relationships or finding individual champions who can help make introductions can be particularly important. As part of trust building, it remains important to bring the findings back to the community, CBOs, and other people well-positioned to lead decision-making and effect positive change.

As the partnerships start, it is essential to develop principles for engagement, and to provide robust incentives for participation for community partners and those involved in data collection. It is also important to keep in mind that in order to provide feedback, the community needs ample time to review instruments and to reflect on the data. If community members or staff workers are performing interviews, having solid training in place and giving interviewers the opportunity to process primary or secondary trauma is essential. Many of the interviewers for this study were people who also live with a disability; and, on reflection, they could have re-experienced some of their own trauma and/or secondary trauma hearing about the experiences from the interviewees. Making sure to provide interviewer training around recognizing secondary trauma, providing safe places to share and process, and possibly having longer times between interviews to give interviewers a chance to decompress should be some of the built-in safeguards.

This study has built on the existing literature by examining various types of social, economic, and health impacts of COVID-19 on people living with disabilities, and how the available supports helped alleviate them. Future research should include deeper dives into this population, particularly with a larger sample size to understand both the specific disability type experiences and the effects of intersectionality of race/ethnicity, income, and disability type. Given the length of this public health emergency, considering other mechanisms for data collection, such as panel surveys, may allow for a more real-time understanding of how the policy changes are impacting the population of people living with disabilities, to allow policymakers to obtain actionable information to address community needs.

## 5. Conclusions

The findings show that people living with disabilities experienced some positive outcomes from the COVID-19 pandemic, but the impacts were mostly negative. The study also indicates that connections to supports, such as financial stimulus, food stamps, and mental health resources, were beneficial to this community and provided some measure of amelioration of negative impacts. There are key lessons that the policy makers, public health leaders, and community can take from this process, such as making sure to engage with this vibrant and important community. The community of people living with disabilities would benefit from specialized attention when planning for public health responses, especially when the needs of the community are diverse. Finally, Public Health should advocate for systematic data collection for routine datasets to draw attention to the inequities faced by those living with disability, in order to develop policies and practices that address these issues.

## Figures and Tables

**Table 1:** PHSKC framework for social, economic, and overall health outcomes.

Topic Area	Indicator
Economic	Unemployment claims
	Social service needs
	Traffic, transportation
Social	Food insecurity
	Family violence
	Access to internet and technology
	Social impacts on childcare, work, ability to stay at home
Health	Access to care
	Mental and behavioral health
	Changes to death patterns
Policy	Federal, state, county, city policies pertaining to COVID-19

**Table 2:** Phase II participant demographics.

Demographics	%	*n* = 35
Age group		
18–24	9%	3
25–34	40%	14
35–44	6%	2
45–54	14%	5
55–64	11%	4
65+	20%	7
Gender *		
Female	63%	22
Non-binary/genderqueer	23%	8
Male	11%	4
Transgender/other	11%	4
Disability type *		
Mental health condition (depression, anxiety, bipolar, schizophrenia, etc.)	91%	32
Other disability or chronic condition (dyslexia, HIV/AIDS, cancer, diabetes, etc.)	83%	29
Developmental or intellectual disability (Down syndrome, Autism, ADHD, etc.)	54%	19
Mobility disability (use a wheelchair, walker, cane, prosthetic, etc.)	43%	15
Sensory disability (blindness, low-vision, d/Deaf, hard-of-hearing, Deaf, Blind, etc.)	8%	8
Race/ethnicity *		
American Indian/Alaska Native	9%	3
Asian	14%	5
Black	23%	3
Hispanic	3%	1
Native Hawaiian/Pacific Islander	6%	2
White	66%	23
Another race/ethnicity	6%	2
Prefer not to answer	6%	2
Previously tested positive for COVID		
Yes	20%	7
No	80%	28
Vaccination status		
Fully vaccinated and boosted	69%	24
2 shots only	23%	8
Not vaccinated	9%	3

* Categories are not mutually exclusive, so percentages will add up to more than 100%.

## Data Availability

Not applicable.

## Supplementary Materials

The following supporting information can be downloaded at: https://www.mdpi.com/article/10.3390/ijerph191710520/s1, File S1: Confidentiality Agreement Interviewers; File S2: Protocol Listening Session with CBOs; File S3: Protocol Listening Session with KCDC; File S4: Data collection tool for interviews.
