# Supplementary material for: Social, Economic and Overall Health Impacts of COVID-19 on People Living with Disabilities in King County, WA
Source: Int J Environ Res Public Health. 2022 Aug 24;19(17):10520. doi: 10.3390/ijerph191710520 (PMC9517928; doi:10.3390/ijerph191710520)
Supplement: Supplementary file 1 [file ijerph-19-10520-s001.zip › File S1_ Confidentiality Agreement Interviewers.pdf]

**Confidentiality Agreement**

This agreement is between:

Amy Laurent, Public Health Seattle and King County (Researcher)

and

___________________________________________ (interviewer)

For Social, Economic and Overall Health Impacts of COVID Mitigation Efforts Evaluation (CK19-1904)

Overview of work description: Conduct and record interviews for people living with disabilities about their experiences during the pandemic.

I, interviewer, agree to:

1. keep all the evaluation information shared with me confidential. I will not discuss or share the research information with anyone other than with the Researcher(s) or others identified by the Researcher(s).
2. keep all evaluation information secure while it is in my possession.
3. return all evaluation information to the Researcher(s) when I have completed the tasks or upon request, whichever is earlier.
4. destroy all evaluation information regarding this research project that is not returnable to the Researcher(s) after consulting with the Researcher(s).
5. comply with the instructions of the Researcher(s) about requirements to physically and/or electronically secure records (including password protection, file/folder encryption, and/or use of secure electronic transfer of records through file sharing, use of virtual private networks, etc.).
6. not allow any personally identifiable information to which I have access to be accessible (unless specifically instructed otherwise in writing by the Researcher(s)).

Interviewer:

| | | |
|---|---|---|
| ____________________________ | _____________________________ | ________ |
| *(Print name)* | *(Signature)* | *(Date)* |
