# Supplementary material for: Social, Economic and Overall Health Impacts of COVID-19 on People Living with Disabilities in King County, WA
Source: Int J Environ Res Public Health. 2022 Aug 24;19(17):10520. doi: 10.3390/ijerph191710520 (PMC9517928; doi:10.3390/ijerph191710520)
Supplement: Supplementary file 1 [file ijerph-19-10520-s001.zip › File S2_ Protocol Listening Session with CBOs.pdf]

King County

Impact of COVID-19 and mitigation measures on people living with disabilities

Listening Session with CBOs

Protocol

Background and objective

Public Health Seattle & King County (PHSKC) has been conducting a Social, Economic, and Overall Health evaluation that looks at COVID-19 and the mitigation strategies taken to slow its spread in King County. Most of the data sources that PHSKC uses to monitor key economic, social and health indicators unfortunately do not include data disaggregated by disability status. For that, we have partnered with the Centers for Disease Control and Prevention (CDC) to fill in this gap in the data and provide insights on how COVID-19 has been impacting people with disabilities.

Data collection questions

1. What are the main impacts of the COVID-19 pandemic and the measures put in place to address it (e.g. stay-at-home orders, social distancing etc) on people living with disabilities?
   - What are the impacts in terms of employment/work?
   - What are the impacts in terms of housing?
   - What are the impacts in terms of food security?
   - What are the impacts in terms of mental/behavioral health?
   - What are the impacts in terms of physical health?
   - Are there any other impacts in addition to the ones we talked about?

2. In what ways do you think COVID-19 impacts were different for people living with disabilities, if at all?
3. How are these impacts different for people living with different types of disability?
4. What would have been helpful to support the population you serve to deal with the impacts of COVID-19?
5. How is vaccination hesitancy amongst the population you serve? What are the concerns folks have about getting a COVID-19 vaccine?
   a. What would be helpful to support folks to get vaccinated?
