# Supplementary material for: Social, Economic and Overall Health Impacts of COVID-19 on People Living with Disabilities in King County, WA
Source: Int J Environ Res Public Health. 2022 Aug 24;19(17):10520. doi: 10.3390/ijerph191710520 (PMC9517928; doi:10.3390/ijerph191710520)
Supplement: Supplementary file 1 [file ijerph-19-10520-s001.zip › File S4_ Data collection tool for interviews.pdf]

King County

Impact of COVID-19 and mitigation measures on people living with disabilities: the interview

Introduction

*** Introduction, read by interviewer ***

Thank you for agreeing to talk with us today. My name is... (*Interviewer introduction*).

Your interview will help the local Department of Public Health (Public Health - Seattle & King County) to understand the impacts of COVID-19 on people with disabilities. I will ask you some questions with yes/no or a list of answers and then ask for you to tell me more if you would like. I will be recording your answers.

All of your answers will be private and grouped with all the other interviews. There will be no way to identify any of your personal information. You don't have to answer the questions if you don't want to, and you can ask me to stop at any time. Some questions may feel personal, but the goal is to understand the impacts of COVID-19 for the community overall. Your participation or answers will not negatively impact any services you may receive.

Public Health will share results for comment and review with community before reporting the final report to local, state, and federal policy makers and the Centers for Disease Control and Prevention (CDC). The goal is to inform future policy and elevate the voices of your community.

The conversation should take less than an hour. You will receive $50 for your participation.

Before we begin, I'd like to confirm: is it okay with you that we record this session?

- Yes, Interviewee gave permission to record
- No, Interviewee did not give permission to record

Thank you for your time. This survey needs to be conducted using video and/or audio recording. Without permission to record, the survey will end now.

- Interviewee chooses to terminate survey
- Interviewee chooses to continue participating

****Do Not Read Aloud****

For interviewer to answer:

Are you interviewing the person directly or through a caretaker?

- I am interviewing the person directly
- I am interviewing them through a caretaker

Work/Employment

Q1. I am going read a list of ways that you may get money. What is your current source of income?

(You may select all that apply.)

- I work for others (full time)
- I work for others (part time)

- I work for myself (self-employed)
- I don't work but I am looking
- I don't work and I am NOT looking
- I am unable to work
- I receive benefits: SSI/SSDI (Social Security/Disability)
- I receive benefits: TANF (Temporary Assistance for Needy Families)
- I receive benefits: Pension/Retirement
- I receive benefits: VA Benefits (Military or Veteran's Assistance)
- I receive benefits: Other (please specify type of benefits received by recording or typing response in the text box above)
- Prefer not to answer this question

(ask if Q1=other response) Q1a. Please specify your other source of income.

Q2. Has the COVID-19 pandemic impacted your work situation?

- Yes
- No
- Prefer not to answer this question

(ask if Q2=yes) Q2a. How has the COVID-19 pandemic impacted your work situation? I'll read a list where you can select all that apply and then invite you to tell me more.

> *Interviewer prompts:*
> *Do you mind sharing more about how the pandemic influenced your work?*
> *How did pandemic affect your work or employment?*

- lost my job, and am not looking for another right now
- lost my job, and have not found another one yet
- lost my job, and got a new one
- got furloughed (laid off, temporarily) or had work hours cut
- got a pay (or hours) cut
- sought out new or additional sources of income
- got a new job
- stopped working to care for self or others in the household
- my workplace closed due to COVID
- Other situation (Please click "Record" or enter response in the text box above)
- Prefer not to answer this question

Finances

Q3. As a result of the COVID-19 pandemic, have you or your household been impacted financially in any way?

- Yes
- No
- Prefer not to answer this question

(ask if Q3=yes) Q3a. How have you or your household been impacted financially as a result of the COVID-19 pandemic? I'll read a list and then invite you to tell me more.

> *Interviewer prompts:*
> *How else did the pandemic affect your finances?*

Could you tell me more?

- delayed paying my rent/mortgage
- delayed paying other bills
- cut back on savings
- accumulated more debt than normal (had more/higher bills than normal)
- lost access to my health insurance
- did not have enough money to buy food (on one or more occasions)
- did not have money to pay for my medication/treatment
- provided financial support for family member or friend
- Other (Please click "Record" or enter response in the text box above)
- Prefer not to answer this question

Q4. Since the pandemic started (February 2020), have you had a time where the food that you bought just didn't last, and you didn't have money to get more?

- Yes
- No
- Prefer not to answer this question

(ask if Q4=yes) Q4a. Prior to the pandemic (before February 2020), had your household had a situation where the food you bought just didn't last, and you didn't have money to get more?

- Yes
- No
- Prefer not to answer this question

Q5. Since the pandemic started (after February 2020), did anyone in your household ever eat less than they felt they should because there wasn't enough money to buy food?

- Yes
- No
- Prefer not to answer this question

(ask if Q5=yes) Q5a. Prior to the pandemic (before February 2020) did anyone in your household ever eat less than they felt they should because there wasn't enough money to buy food?

- Yes
- No
- Prefer not to answer this question

Q6. Have you received funds from a relief program related to COVID-19 (e.g. utility discount, rent assistance)?

Interviewer prompt:
Please tell me more about the assistance you received or why you weren't able to get what you needed?

- No – I didn't need it
- No – I didn't know about these resources
- No – I didn't know how to apply
- No – I applied but did not receive (e.g. did not meet the criteria)
- Yes – I applied by myself
- Yes – A third party helped me (i.e. friend, family member, CBO)
- Prefer not to answer this question

Housing

Q7. What is your current housing situation? I will read you a list; please let me know which applies to you.

- I live in a home that I rent (or someone in my household rents)
- I live in a home that I own (or someone in my household owns)
- I live in a home I share with others, but do not own/rent
- I live in a shelter
- I couch surf (staying with friends/family for short periods of time)
- I sleep in a car/tent
- Other (Please click "Record" or enter response in the text box above)
- Prefer not to answer this question

Q8. Has the COVID-19 pandemic impacted your housing situation?

- Yes
- No
- Prefer not to answer this question

(ask if Q8=yes) Q8a. How has the COVID-19 pandemic impacted your housing situation? I will read a list and then invite you to tell me more.

> *Interviewer prompt:*
> *How did this affect you?*

- delayed paying my rent/mortgage
- had to apply for rent assistance programs
- had to move to a different home
- chose to move to a different home
- became homeless
- Other (Please click "Record" or enter response in the text box above)
- Prefer not to answer this question

Mental/Behavioral health

Q9. Did your mental/behavioral health change since the beginning of the COVID-19 pandemic (February 2020)?

- Yes
- No
- Prefer not to answer this question

(ask if Q9=yes) Q9a. How did the COVID-19 pandemic impact your mental/behavioral health? I will read a list and then invite you to tell me more.

We also have a list of resources, if that would be helpful, that I can provide you with after the interview.

> *Interviewer prompts:*
> *Do you mind sharing more about the pandemic's effect on your mental health?*
> *What about the pandemic affected your mental health?*
> *Do you still feel this way?*
> *Were there things that helped the situation?*

- have felt anxious or depressed more often
- have felt anxious or depressed less often

- started having less healthy habits (e.g. eating too much, smoking, drinking, using drugs or other substances)
- started having healthier habits (e.g. eating better, exercising, more time with hobbies)
- lost access to counselor/support system
- increased access to counselor/support system
- did not have continued access to necessary medication for mental health
- other (Please click "Record" or enter response in the text box above)
- Prefer not to answer this question

Physical Health

Q10. Have you or anyone you live with been diagnosed as having COVID-19? (At any time since the pandemic started)

- Yes, I have
- Yes, someone I live with
- Yes, both myself and at least one other person I live with
- No one in my household or where I live
- Prefer not to answer this question

(ask if Q10=yes) Q10a. After having COVID-19, have you had any lingering side effects (also known as “long COVID”)?

- Yes
- No
- Prefer not to answer this question

(ask if Q10a=yes) Q10b. What were these side effects or symptoms of long COVID? I'll list out some symptoms and please let me know all that apply.

- Chest Pain
- Cough
- Depression or Anxiety
- Difficulty thinking, concentrating, or remembering things (sometimes referred to as "brain fog")
- Dizziness on standing
- Fast-beating or pounding heart (also known as heart palpitations)
- Fever
- Headache
- Joint or muscle pain
- Loss or change of smell or taste
- Shortness of breath
- Tiredness or fatigue
- Trouble sleeping
- Symptoms that get worse after physical or mental work
- Prefer not to answer this question

Q11. Since the COVID-19 pandemic started, have you...

Interviewer prompt:
Could you tell me more about how this impacted you?

- avoided seeking health care because of concern about getting COVID-19?
- had surgery or other medical procedure delayed because of the pandemic?

- had any other impacts on your physical health due to the pandemic? (please record or type response above)
- Prefer not to answer this question

General Impact of COVID-19

Q12. We asked you about the impacts of COVID-19 on your work and finances, your housing situation, and your health. Are there any other ways that the pandemic has impacted your life that you would like to share?

Vaccination

Q13. Have you received the COVID-19 vaccine?

- Yes, I'm partially vaccinated
- Yes, I have had 2 doses
- Yes, I have had 2 doses and least 1 booster dose
- No
- Prefer not to answer this question

(ask if Q13=no or vaccination partial/two doses) Q13a. Could you talk a bit more about why you didn't get vaccinated/ did not complete the vaccination / did not take a booster? I'll read a list and then invite you to tell me more.

Interviewer prompts:
Do you mind sharing any worries or thoughts you have?
Could you tell me more about your response?

- I haven't been able to get a COVID-19 vaccine
- I do not plan to get a COVID-19 vaccine
- I am not sure if I will get the COVID-19 vaccine
- I am unable to get a COVID-19 vaccine due to my medical condition
- I am not sure I want a booster
- Prefer not to answer this question

Health Insurance

Q14. Do you have health insurance? Please select all that apply

- No
- Yes – Apple Health/Medicaid
- Yes – Medicare
- Yes – Tricare (VA)
- Yes – Health insurance through employer
- Yes – Health insurance purchased privately
- Yes – Other, please specify (Please click "Record" or enter response in the text box above)
- Prefer not to answer this question

Disability(ies)

Q15. Do you experience any of the following? Please select all that apply

- Developmental or intellectual disability (Down syndrome, Autism, ADHD etc.)
- Mental Health condition (depression, anxiety, bipolar, schizophrenia, etc.)
- Mobility disability (use a wheelchair, walker, cane, prosthetic, etc.)
- Sensory disability (blindness, low-vision, d/Deaf, hard-of-hearing, DeafBlind, etc.)

- Other disability or chronic condition (dyslexia, HIV/AIDS, cancer, diabetes, etc.)
- Prefer not to answer this question

(ask if Q15 is not prefer not to answer/not answered) Q16. How much would you say your disability(ies) impacts your ability to perform daily activities?

- Insignificant impact
- Minor impact
- Moderate impact
- Major impact
- Severe impact
- Prefer not to answer this question

Q17. When thinking about the COVID-19 response, what do you think should be done differently for future pandemics?

Q18. Is there anything else about the COVID-19 pandemic you'd like to share with us?

Interviewer prompt:
If you could tell the government anything about the pandemic, what would you tell them?

Demographics

We have a few more questions about demographics that will help us better frame your experiences.

Race/ethnicity

Add all that apply

- American Indian or Alaska Native
- Asian Indian
- Chinese
- Filipino(a)
- Japanese
- Korean
- Vietnamese
- Other Asian (please add in the box above)
- African American
- Somali
- Ethiopian
- Other Black, African, or African American (please add in box above)
- Mexican, Mexican American, Chicano
- Cuban, Dominican Republican or Puerto Rican
- Central American
- Other Hispanic, Latino(a)/Latinx or Spanish
- Middle Eastern or North African
- Native Hawaiian
- Samoan
- Other Pacific Islanders
- White
- Other race (please add in box above)

- Prefer not to answer

Gender identity

- Female
- Male
- Nonbinary or genderqueer
- Transgender
- Other
- Prefer not to answer

Sexual orientation

- Bisexual or pansexual
- Gay, lesbian, or homosexual
- Heterosexual or straight
- Queer
- Other
- Prefer not to answer

Age group

What is your current age (in years)?

- 18-24
- 25-34
- 35-44
- 45-54
- 55-64
- 65 or older
- Prefer not to answer

What is the ZIP code where you currently live?

(99999 - unhoused or if without a fixed address; note if prefer not to answer)

Final message

Thank you for taking the time to participate in this interview and for sharing your experiences. We will compile all the responses for a comprehensive report and give community an opportunity to review and provide feedback before we share the findings with policy makers at the local, state, and federal level. If you would like to be notified when the report is ready for community review, please share your e-mail or phone number so we can let you know. (we will remove the email/phone number once stored so it is not associated with your responses)

For compensation, please talk with your interviewer about their process

If interviewee would like information about results, please add their email or phone number.

Thank you!

Phone number or e-mail (please verify)

Interviewer questions

These next questions are for the interviewers to complete post-survey

Interviewers: please enter your initials

Was this interview conducted in a language other than English?

- Yes
- No

In what language was the interview conducted?

- Spanish
- ASL (American Sign Language)
- Other (please enter into the text box under the Record button)

Interviewers: are there any thoughts you want to share about this interview? (e.g. did it end early; did the interviewee have trouble with specific questions)

Thank you. Please remember to save the Zoom or recorder recording to the MS Teams site. The information for this interview is complete.
